# New *Corynebacterium* Species with the Potential to Produce Diphtheria Toxin

**DOI:** 10.3390/pathogens11111264

**Published:** 2022-10-30

**Authors:** Marta Prygiel, Maciej Polak, Ewa Mosiej, Karol Wdowiak, Kamila Formińska, Aleksandra Anna Zasada

**Affiliations:** Department of Sera and Vaccines Evaluation, National Institute of Public Health NIH, National Research Institute, 00-791 Warsaw, Poland

**Keywords:** NTTB, diphtheria toxin, *Corynebacterium*, *tox* gene, *C. rouxii*, *C. silvaticum*, *C. belfantii*, *C. diphtheriae*, *C. ulcerans*

## Abstract

Only three *Corynebacterium* species are known to produce a lethal exotoxin called diphtheria toxin. These are *C. diphtheriae, C. ulcerans* and *C. pseudotuberculosis*. The diphtheria toxin gene (*tox*) is carried in a family of closely related corynebacteriophages and therefore the toxin can be produced only through lysogenisation, in which the corynephage encoding *tox* is stably inserted into the chromosome. However, ‘nontoxigenic *tox* gene-bearing’ (NTTB) strains, which are genotypically *tox*-positive but do not express the protein, have been described. The emergence of NTTB strains was first observed during the 1990s diphtheria epidemic in Eastern Europe and nowadays such isolates have been detected in many countries in the world. Recently, novel species of *Corynebacterium* genus have been described which might have the potential of producing the diphtheria toxin due to the possession of the diphtheria toxin gene but it has not produced toxin in laboratory tests. The circulation of NTTB strains could be related to the increased risk for diphtheria disease arising from the risk of re-emerging toxin expression. The article presents the mechanism of diphtheria toxin expression and action, recently described novel species of NTTB corynebacteria as well as the taxonomic changes within the *C. diphtheriae* group.

## 1. Introduction

The genus *Corynebacterium* was first described in 1896 as a Gram-positive club-shaped bacillus with filamentous morphology [1]. The genus *Corynebacterium* belongs to the Phylum Actinobacteria characterized by high cytosine and guanine contents in DNA. Currently, this genus included about 145 different species [2]. More than half of the species were isolated from human and animal clinical samples which indicates their potential participation in pathogenesis [1,2]. In addition, strains of medical and veterinary importance, of the same species, such as *Corynebacterium glutamicum* and *Corynebacterium efficiens*, have biotechnological applications [1,2]. 

The most important human pathogen is *Corynebacterium diphtheriae*, which is the etiological agent of diphtheria, a serious, potentially fatal infection of the respiratory tract and occasionally the skin and other mucous membranes such as, e.g., eye, ear, and genital tract. The infection often causes complications in other body organs [3]. Since Friedrich Löffler’s isolation of toxin-secreting *C. diphtheriae* in 1884 [1], the species has been the best-known and probably most genetically diverse species of the genus [4,5]. Classical diphtheria is caused by the production of diphtheria toxin (DT) during infections by isolates holding the toxin gene. DT is the main virulence factor responsible for respiratory, neuro- or cardiopathological symptoms, causing pseudo-membranes, paralysis and cardiac failure [1]. In countries with high anti-diphtheria vaccination coverage, the disease is very rare, but in some regions of Africa and Asia diphtheria is still recognised, with thousands of cases reported annually [3]. The disease can emerge in case of the failed implementation of the recommended vaccination programs or lack of booster doses [3]. 

*Corynebacterium ulcerans* was described in 1926 by Gilbert and Steward [6]. The species is closely related to *C. diphtheriae* and also is able to produce DT. Nowadays, in European countries, *C. ulcerans* is recognized more frequently as an emerging pathogen associated with diphtheria-like symptoms [7,8]. Growing numbers of human infections caused by *C. ulcerans* are the result of zoonotic transmission by contact with animal hosts such as goats, cattle, domestic pigs, dogs, cats and even hedgehogs, monkeys, camels, foxes, squirrels, owls, orcas, otters and water rats [9].

*Corynebacterium pseudotuberculosis* is the etiological agent of ulcerative lymphangitis in equines, mastitis in dairy cattle, oedematous skin disease in buffalos, or abscesses and caseous lymphadenitis (CLA) in small ruminants, such as goat and sheep [10,11]. *C. pseudotuberculosis* has caused occasional infection in farm and animal health workers who remain in close contact with infected animals or their raw products, resulting in swellings of the lymph nodes in the neck or groin. *C. pseudotuberculosis* animal diseases cause severe economic losses [12]. The bacterium was first described in 1888 by Edmond Isidore Etienne Nocard and classified as *C. pseudotuberculosis* in 1918 by Eberson [13]. Historically, it is the third species known to be able to produce DT. However, toxin-producing *C. pseudotuberculosis* has been isolated extremely rarely. 

Among the pathogenic species of the genus *Corynebacterium*, the *C. diphtheriae, C. ulcerans* and *C. pseudotuberculosis*, which can produce DT, were clustered together in the group of toxigenic corynebacteria named “*C. diphtheriae* complex” [14]. 

However, the infections caused by potentially toxigenic corynebacteria have recently changed. Toxigenic *C. ulcerans* has been isolated from clinical samples more often than in preceding years. Serious invasive infections caused by nontoxigenic *C. diphtheriae* have been noticed in many countries with high anti-diphtheria vaccination coverage [15]. What is more, new species capable of producing DT were described in 2020, called *C. rouxii* and *C. silvaticum* [16]. Based on the genomic sequencing and biochemical and chemotaxonomic analyses, the name of *C. belfantii* was proposed for strains previously considered *C. diphtheriae* biotype *belfanti* [17]. In this paper, we present current information concerning DT and potentially toxigenic *Corynebacterium* species. 

## 2. The Structure of Diphtheria Toxin

Diphtheria toxin (DT) is encoded by a 1683-base-pair structural gene-*tox* (NCBI Reference Sequence: NC_002935.2), encoded not on the bacterial chromosome, but by a lysogenic phage called corynebacteriophage beta or corynephage β [18,19]. The integration of the corynephage β can occur at two specific sites: *attB1* and *attB2* [20]. Although *tox* is of bacteriophage origin, the regulation of its expression is reliant on bacteria [21]. The diphtheria toxin repressor gene (*dtxR*) is present on the bacterial chromosome. Its protein product (DtxR) is able to bind to the *tox* operator, blocking the transcription [22,23]. Interestingly, DtxR is activated by heavy metals, especially iron ions [23,24,25]. In the absence of iron ions apo-DtxR exists as an inactive monomer that is in weak equilibrium with a dimeric form. Once activated by the metal ions, DtxR forms stable dimers and two pairs of dimers have been shown to bind to almost opposite faces of the *tox* operator sequence. DtxR is composed of two major structural domains linked by a flexible tether containing a proline-rich region. The N-terminal domain contains the ancillary and primary metal ion-binding sites, a canonical helix-turn-helix DNA-recognition motif, and an extensive hydrophobic surface necessary for the formation of stable dimers. After DtxR dimers binding to *tox* promoter, the transcription of *tox* is repressed. When iron is limiting, the uncomplexed form of DtxR is unable to bind DNA, leading to the induction of diphtheria toxin [20,21]. The mature extracellular DT is a polypeptide consisting of 535 amino acid residues with a molecular mass of approximately 58 kDa [26,27,28]. The gene sequence analysis indicates that DT is preceded by 25 residues of leader peptide, which is most likely involved in toxin secretion [19]. DT is produced as a proenzyme that requires specific activation for its toxic function, either prior to or immediately after binding to a sensitive cell [29]. It is based on a proteolytic cleavage which cleaves the peptide bond located at the arginine (Arg) residue: Arg190, Arg192, or Arg193, resulting in the formation of two polypeptides: (1) a 193-residue amino-terminal fragment A (DT-A) which corresponds with the catalytic domain of DT, and (2) a 342-residue carboxyl-terminal fragment B (DT-B), corresponding with the translocation and receptor-binding domains of DT [26,30]. Both fragments remain covalently bound by the disulphide bonds between Cys186 and Cys201, and a reduction of this binding results in free forms of DT-A and DT-B, capable of infecting target cells [27]. DT has been described as the first example of group A–B toxins in which the catalytic and receptor-binding functions are separated into two different polypeptides [31]. Currently, the A–B motif is well-known and almost universal among intracellular toxins [32,33].

**Figure 1:**
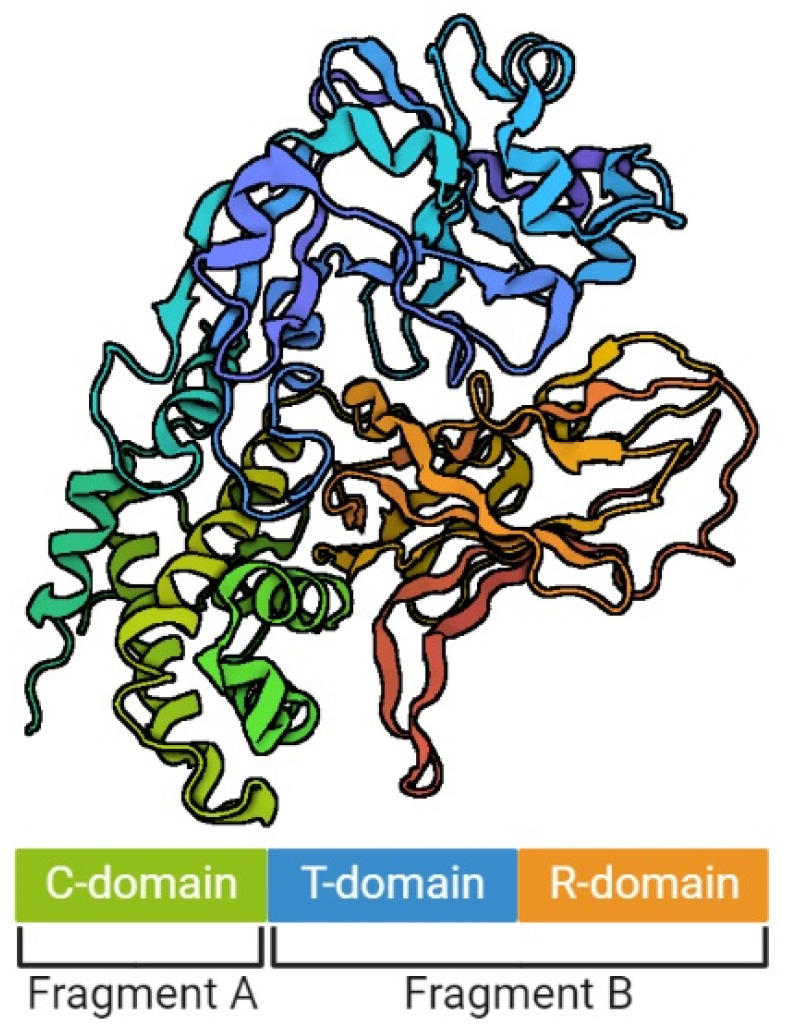
The structure of diphtheria toxin. The figure was created with BioRender.com, accessed on 1 September 2022.

The model of DT structure has evolved over the years, along with the methods available for its determination [29,34,35]. It is assumed that a single DT molecule has three distinct folding domains – C, T and R (Figure 1), symbols of which are derived from the three main functions of this toxin, respectively: catalysis, translocation and receptor binding, respectively. They are arranged in the shape of the letter Y, with the lower part being the T domain, and the upper elements consisting of the C and R domains. The T domain is formed by the α-helical bundle, the R domain by the flattened β-barrel, and the C domain in turn is a combination of structures α and β. Functionally, the C domain forms fragment A of the mature DT, and the T and R domains – fragment B [29,35,36]. Concurrently, a crystallographic analysis showed that in the discussed Y shape, the cleft in the active site of the C domain is blocked by the R domain from accessing the substrate. This is the reason why those two fragments must be separated from each other in order to be toxic [29]. However, no trypsin-sensitive loop was found between the C and T domains that might be responsible for proenzyme proteolysis. Probably this place is created dynamically, which makes detecting it impossible [31]. Furthermore, there was an emphasized high similarity of the T domain to the hydrophobic N-terminal domain of the B chain of the non-toxic protein CRM45, that the ability to form pores in membranes under acidic conditions was attributed to [37,38].

## 3. The Mechanism of DT Toxicity 

DT toxicity towards sensitive cells is based on the inactivation of a protein elongation factor-2 (EF-2), which is an essential element for protein synthesis, stimulating the GTP-dependent translocation of the ribosome [39,40,41]. This death process begins with the binding of DT by a receptor—a membrane-anchored form of the heparin binding EGF-like growth factor (HB-EGF precursor), on the cell surface of DT-sensitive cells. DT enters the cytosol via receptor-mediated endocytosis [28,42,43]. Inside endosomes, proteases partially cleave the bond between the A and B fragments of the toxin. The low pH inside the endosomes promotes a conformational change in DT by which the T domain is inserted into the endosomal membrane thereby allowing cytosolic DT-A exposure [44]. Then, the specific toxicity reaction begins—the A fragment transfers the ADP-ribose moiety of nicotinamide adenine dinucleotide (NAD^+^) to a modified histidine residue (diphthamide) on EF-2, deactivating it. Thus, the host is unable to produce protein and dies [39,45]. DT-A can exhibit its toxic activity against all eukaryotic EF-2s except mice and rats, and only one molecule of the toxin in the cytosol is sufficient to lead to cell death [37,46,47].

## 4. Nontoxigenic Toxin Gene-Bearing Strains

Historically, among the *C. diphtheriae* species, toxin-producing and non-toxin-producing strains have been distinguished. However, there has currently also been a third category: ‘nontoxigenic *tox* gene-bearing’ (NTTB). Non-toxin-producing (nontoxigenic) *C. diphtheriae* strains generally do not possess the *tox* gene, with the exception of some nontoxigenic strains which bear the *tox* gene. These strains called ‘nontoxigenic *tox* gene-bearing’ (NTTB) are genotypically *tox*-positive, but do not express the diphtheria toxin due to nucleotide mutations or deletions [48]. The circulation of NTTB *C. diphtheriae* strains was first detected during and after the diphtheria epidemic in the 1990s in Belarus [49] and in Russia during 1994–2002 [50]. NTTB *C. diphtheriae* strains have also been reported in other regions, for example in the United Kingdom [48,51], the United States [52] and Australia [53].

It is likely that mutations causing *tox* gene inactivation might be frequent in *C. diphtheriae* after epidemic waves, as a result of pathogen adaptation to circulation in the population with high anti-diphtheria antibody levels [54]. 

Nontoxigenic *C. diphtheriae* is recognized as a potential emerging pathogen, as it is with increasing frequency associated with severe invasive diseases. The growing number of detected nontoxigenic *C. diphtheriae* infections in the 1990s and in the early 2000s points out that the circulation of these strains is an escalating problem in Europe [55,56,57,58,59,60,61]. Nontoxigenic *C. diphtheriae* infections cannot be prevented by the contemporary vaccines targeting diphtheria toxin [62,63,64] and have quickly become prevalent in countries with high anti-diphtheria vaccination coverage. 

Due to the fact that only toxigenic infections must be reported, the extent of the problem of nontoxigenic *C. diphtheriae* infections in Europe remains unknown since only toxigenic infections are registered. The lack of mandatory registration consequently leads to no prevention measures, which results in the spread of strains able to cause infections [65]. Nontoxigenic *C. diphtheriae* strains may become a public health threat in developed countries because they can persist and spread in the risk groups and then become the source of an outbreak [55].

The pathogenesis of nontoxigenic *C. diphtheriae* is not well elucidated. The most likely entry portals for nontoxigenic *C. diphtheriae* are skin lesions or dental caries [65]. In recent years, severe and often fatal systemic diseases (which were previously quite rare) caused by nontoxigenic *C. diphtheriae* have been registered in various countries. Nontoxigenic *C. diphtheriae* often were found to be associated with cutaneous lesions but can transform into severe clinical symptoms, such as myocarditis, polyneuritis, bacteraemia, septic arthritis and endocarditis, characterized by a high mortality rate reaching over 40% [58,60,61,63,65]. Among the factors that predispose to the invasive infections caused by nontoxigenic *C. diphtheriae* occurrence are homelessness, abuse of alcohol and injection drugs and diabetes mellitus, hepatic cirrhosis and dental caries [61]. Furthermore, refugees and foreign travellers constitute population groups that are particularly at risk of nontoxigenic *C. diphtheriae* infections [66,67]. 

The increasing number of cases of invasive infections caused by nontoxigenic isolates might suggest the acquisition of additional virulence factors [60]. It is theorized that the success in the prevention of toxin-mediated disease as an effect of anti-diphtheria vaccination has created selective pressure on *C. diphtheriae* strains to express or develop disease-causing mechanisms and virulence factors other than diphtheria toxin [60]. 

Nontoxigenic *C. diphtheriae* is a public health concern due to the lack of protection provided by current diphtheria vaccines and the potential for such circulating strains to readily become toxigenic through lysogenisation by toxin-encoding bacteriophages [68].

The knowledge of the potential for nontoxigenic isolates to regain toxigenicity is limited. There is a probability that the integration of *tox*-carrying bacteriophages into the genome of nontoxigenic strains can convert them into toxigenic and virulent strains [48,69]. 

## 5. *Corynebacterium silvaticum*

*Corynebacterium silvaticum* sp. nov. is a novel species of the nontoxigenic *tox*-gene-bearing (NTTB) strains, firstly isolated from lymph nodes of wild boars showing severe lesions due to caseous lymphadenitis (CLA). The first case report on the isolation of atypical *Corynebacterium* strains from two wild boars causing CLA was published in 2011 [70]. The wild boars came from different provinces in southern Germany. Isolated strains (named KL0182T and KL0183) were positive for phospholipase D—the major virulence determinant of *C. pseudotuberculosis*, which plays a key role in the spread of bacteria from the site of infection to the lymph nodes. However, biochemical studies did not allow to classify them as *C. pseudotuberculosis* species. Sequencing of the *16S rRNA* and *rpoB* genes allowed the classifying of the isolates to the *C. ulcerans* species. The *tox* gene for diphtheria toxin was detected in both isolates. DNA sequencing of the *tox* gene exhibited differences from sequences described for *C. ulcerans* strains and showed higher similarity to *C. diphtheriae*. The expression of diphtheria toxin was not be detected phenotypically. These results have indicated that wild boars could be a reservoir for zoonotic *Corynebacterium*. Since the description of these two isolates, bacteria causing caseous lymph node abscesses in wild boar have been consistently collected [71,72], causing the same difficulties in unambiguous classification as *C. diphtheriae, C. belfantii, C. ulcerans* or *C. pseudotuberculosis*, so far addressed as atypical *C. ulcerans* [70,71,72] or “wild boar cluster” (WBC) of *C*. *ulcerans* [73,74].

Based on the results presented below, Dangel et al. [75] classified those isolates into a novel species named *C. silvaticum* sp. nov. Thirty-four *Corynebacterium* sp. strains were collected from CLA of wild boar (33 isolates) and roe deer (1 isolate) from different regions of Germany between 1997 and 2018. Isolation procedures based on microbiological methods have demonstrated that bacteria grow under aerobic and microaerophilic conditions at 37 °C within 48 h on SBA (Sheep Blood Agar) and BHI (Brain Heart Infusion) plates (but slower than other *Corynebacterium* species). Colonies of the bacteria are small, creamy to waxy with a sleek area and a discrete β-haemolysis after incubation. The optimum pH range for growth is 7–8. The bacteria produce phospholipase D, catalase and urease and are inconstant for alpha-glucosidase and alkaline phosphatase production. Isolates show fermentation of ribose, glucose and maltose (same as *C. ulcerans*), but do not metabolize mannitol, D-xylose, lactose, glycogen and sucrose (same as *C. pseudotuberculosis*). They are sensitive to clindamycin, erythromycin and penicillin unlike *C. ulcerans.* The fatty acid analysis has assigned them to the *C. diphtheriae* group of genus *Corynebacterium*. The MALDI-TOF MS and *rpoB* gene sequencing have allocated them to the *C. ulcerans*, but the profile of polar major lipids and glycolipids differs from that of *C. ulcerans.* In the phylogenesis of the 16SrRNA and RpoB proteins, isolates have created separate branches with *C. ulcerans* as the closest relative. The quinone system has been almost identical as in *C. ulcerans* with the main menaquinone MK-8(H2). All analysed isolates (34) have been included in NTTB strains. The presence of the *tox* gene has been confirmed by real-time PCR, but the phenotypic expression of diphtheria toxin has not been detected in the Elek test. Whole genome sequencing has shown the specific sequence type 578 and a separate branch in MLST typing. The average nucleotide identity (ANI) values are <91, draft genome sizes are 2.55 Mbp and the G/C content is 54.4 mol%. Based on these results regarding phenotype, genotype and biochemistry, the said bacteria represent a separate species, for which the authors [75] have proposed the name *C. silvaticum* sp. nov. The full-length *16S rRNA* gene sequence of *C. silvaticum* sp. nov is available at GenBank (accession number MK602323) and WGS draft genomes are available at the NCBI genome database (accession numbers: SDQO00000000, SDVC00000000 and SDVD00000000). The type strain is KL0182^T^ (=CIP 111672^T^ = DSM 109166^T^ = LMG31313^T^) [75]. The study conducted by Möller et al. [76] might indicate a zoonotic potential of *C. silvaticum*. Cytotoxicity of this newly identified species has been demonstrated. This negative influence in in vitro conditions (different human epithelial cell lines) and in in vivo conditions (*Galleria mellonella* larvae) was comparable to diphtheria toxin-secreting *C. ulcerans* [76].

In the first proteome study conducted by scientists [77], 1305 proteins of *C. silvaticum* were identified. The potential known virulence factors such as phospholipase D and sialidase were also detected. Furthermore, an uncharacterized secreted protein trypsin-like protease having an impact on pathogenicity was found. In addition, the said proteome analyses confirmed the taxonomic relationship of *C. silvaticum* to be closely connected with the zoonotic species of the *Corynebacterium* genus.

The results of the study conducted by Viana et al. [78] showed that *Corynebacterium* PO100/5 strain (the first sequenced genome of a *C. silvaticum*) can also colonize livestock and not only wild forest animals. This strain was isolated from a skin abscess taken from a domestic pig in the southern region of Portugal. It was the first strain of *C. silvaticum* isolated outside Germany. The taxonomic analysis revealed that *C. silvaticum* species is genetically more homogeneous than *C. ulcerans*. Moreover, *C. silvaticum* has pilus subunit genes *spaB* (which play important role in adhesion on pharyngeal epithelial cells), conserved genomic islands and 172 genes that could be used as markers for molecular identification [78]. 

Initially recognized as the atypical *C. ulcerans* strains: isolate W25 and isolate KL1196 have been recently isolated from a case of CLA from wild boar and roe deer, respectively [79]. Phylogenetic analyses showed that those strains belong to a novel species *C. silvaticum.* The ANI values between the tested strains were 99% indicating a close relationship to the same species, the ANI values between the tested strains and the *C. ulcerans* genome were 92%, which proves belonging to a different species. The authors proved that one of the key biochemical differences separating *C. silvaticum* from *C. ulcerans* was the inability to ferment starch [79]. 

Wild boars and domestic pigs are reservoirs of *C. silvaticum* which are suspected to transmit the bacteria to other domestic animals and, eventually, humans. Therefore, finding the unique sequence and genes useful for this species classification is crucial for its detection and proper identification. Molecular characterization of *C. pseudotuberculosis, C. auriscanis* and *C. silvaticum* by ERIC 1+2-PCR genotyping could be useful as a diagnostic tool for the detection of the etiological agent of CLA [80]. The study conducted by Ramos et al. [80] showed that ERIC 1+2-PCR genotyping fingerprinted all tested eighty isolates into 24 genotypes: 22 genotypes corresponded to *C. pseudotuberculosis,* 1 genotype to *C. auriscanis* and 1 genotype to tested *C. silvaticum* strains. The maximum genetic similarity of 76% between *C. pseudotuberculosis* and *C. silvaticum* was observed [80]. The number of bands detected for all tested *C. silvaticum* isolates was 13 in the size range from 98 bp to 731 bp. Two of them (475 bp and 426 bp) were peculiar only to *C. silvaticum* profile. This study also confirmed that these bacteria were correctly classified into a new species called *C. silvaticum.*

The presence of mycolic acids in the outer membrane of *the Corynebacterium* genus is an important feature which may be related to virulence [81]. Dover et al. [81] analysed the genomes of 140 corynebacterial strains (representatives of 126 different species), the majority of which were isolated from humans and animals, and presented that these species had been organised into 19 phylogenetic groups proving their great diversity. Most of the important human and animal pathogens have been grouped into one group called Q. The group Q includes *C. diphtheriae, C. pseudotuberculosis, C. ulcerans, C. silvaticum* and *C. rouxii*. This similarity indicates the possibility that animal isolates can infect people [81]. 

## 6. *Corynebacterium rouxii*

*Corynebacterium rouxii sp. nov*. described as the new species of *C. diphtheriae* complex [16], was isolated between 2013 and 2017 in France. The isolates came from infected human skin tissues (ulceration) and peritoneum and from a dog’s skin and were initially identified as *C. diphtheriae* biovar *belfanti*. Previously, some *Corynebacterium* strains were isolated from domestic cats in the United States in 2010 with properties similar to the novel species *C. rouxii* [82]. Nowadays, after the proper species classification, those strains described in the United States have been reclassified as a novel species, *Corynebacterium rouxii sp. nov.*

*C. rouxii* is biochemically similar to *C. diphtheriae* biovar *belfanti* (currently reclassified as *C. belfantii*) except that the strains are negative for maltose fermentation. The investigated isolates were trehalose, urease, pyrazinamide, nitrate and glycogen negative. Moreover, all the investigated isolates of *C. rouxii* were positive in the PCR test for *dtxR* and *rpoB* genes and negative for *C. ulcerans/C. pseudotuberculosis* 16S rDNA and *pld* which indicated a similarity to *C. diphtheriae* but not to *C. ulcerans* and *C. pseudotuberulosis*. The *tox* gene was not detected [83,84]. 

The average genome size of *C. rouxii* is 2.4 Mb. The genomic sequencing results showed that the ANI value of *C. rouxii* was 92.4% with the *C. diphtheriae* clade and 91.4% with *C. belfantii*. The *C. rouxii* clade was genetically homogenous, which was revealed by ANI values ranging from 99.21% to 99.94% [83]. 

The type strain is FCR0190^T^ (=CIP 111752^T^ = DSM 110354^T^). The genome sequence is available in GenBank under access number MN535983. 

## 7. *Corynebacterium belfantii*

*C. diphtheriae* was historically subdivided into four biovars: Mitis, Gravis, Intermedius and Belfanti, based on the colony morphology and biochemical properties (Table 1) [3,85]. The names of the first three biovars were supposed to refer to the illness severity they cause, however according to the current molecular epidemiology knowledge such a correlation does not occur [86,87]. Biovar Belfanti was later added to highlight the nontoxigenic *C. diphtheriae* strains isolated from ozaena patients that unlike strains from other biovars were nitrate negative [17,88]. This biovar was named after Serafino Belfanti who was the first to identify such strains [17,88]. 

Molecular typing studies have shown that the phenotypical differentiation of *C. diphtheriae* isolates into the biovars does not correspond to their genetic diversity [3,5,87,89,90].The isolates within the same biovar can be genetically more distant than isolates found in different biovars [90]. Recently, changes in *C. diphtheriae* taxonomy have been proposed on the basis of genomic sequencing findings [17,86]. In 2018, Dazas et al. [86] proposed to grant species status *Corynebacterium belfantii* sp. nov. for the group of isolates from the *C. diphtheriae* biovar Belfanti. As they proved, the isolates formed a clearly demarcated branch from *C. diphtheriae* biovars Mitis and Gravis [17]. The average nucleotide identity (ANI) of the *C. belfantii* isolates with *C. diphtheriae* type strain NCTC11397^T^ was 94.85% and below the species threshold for bacteria (~95–96%) [17]. FRC0043^T^ (CIP 111412^T^, DSM 105776^T^) was designated as *C. belfantii* type strain. In another phylogenetic concept, Tagini et al. [86] proposed a subdivision of *C. diphtheriae* into two subspecies—*C. diphtheriae* subsp. *diphtheriae* and *C. diphtheriae* subsp. *lausannense* [86]. However, as noted by Badell et al. [83], *C. diphtheriae* subsp. *lausannense* appears to be a later heterotypic synonym of *C. belfantii*, on the basis of the high genetic similarity between them and publication priority. Shortly thereafter, biovar Belfanti was the subject of another change in the taxonomy of *C. diphtheriae*; it was proposed to classify its atypical strains into a novel species named *C. rouxii* [83].

*C. belfantii* is a human pathogen that is commonly isolated from the respiratory tract, mostly from the nose or throat and often in association with ozaena [91,92]. Isolation of the bacteria from cutaneous infections is extremely rare [91,92,93]. A recent study revealed that *C. belfantii* can colonize and transmit between susceptible patients with cystic fibrosis [91]. Although isolation of *C. belfantii* from animals has been exceptionally rarely reported, such cases must be confirmed to avoid possible misclassification that may have occurred due to recent changes in the taxonomy of *Corynebacterium* genus [83,84,94,95]. In the past, *C. belfantii* species was rarely isolated, and generally in highly vaccinated countries where there is surveillance of *Corynebacterium* spp. infections [89,92]. Recently, with the shift from toxigenic to nontoxigenic *C. diphtheriae* population, the isolation frequency of *C. belfantii* has also increased [89,92]. 

Whole-genomic sequencing studies showed that *C. belfantii* isolates had an average genome size of 2.7 Mb, i.e., larger than that of *C. rouxii* isolates (2.4 Mb) and Mitis/Gravis isolates (2.45 Mb) [17,83]. Although *C. belfantii* is generally considered a nontoxigenic species, the *tox* gene was rarely reported in isolates of the former biovar Belfanti [87,96]. As in the case of *C. diphtheriae*, prophage insertions in the genome of *C. belfantii* are common [93]. For other virulence factors, the classical genes encoding pili (SpaA-, SpaD- and SpaH-type) were non-existent or only one of them (SrtB for SpaD-type pili) was present in *C. belfantii* isolates studied by Tagini et al. [86] and Li et al. [97], respectively. Moreover, in the isolate studied by Li et al. [97] more copies of genes involved in the ABC transporter were found compared to the reference strain of *C. diphtheriae* NCTC 13129, suggesting its potential increase of capacity to uptake iron and nutrition [97].

*C. belfantii* isolates similar to *C. diphtheriae* are generally susceptible to penicillin and erythromycin used for diphtheria treatment [89,92]. However, reduced susceptibility or resistance to one of these antibiotics was reported in some countries [93,97]. Moreover, reduced sensitivity or resistance to ciprofloxacin was described among recently collected isolates, suggesting asymptomatic carrier or undiagnosed *Corynebacterium* spp. infections [89,91,92].

## 8. Conclusions

The first *Corynebacterium* species was described almost 240 years ago. Since then, new species have been discovered and currently, the genus covers approximately 145 species. The development of new microbiological and molecular biology methods enables a more accurate analysis of isolated strains and, consequently, taxonomic changes. On the other hand, the development of sophisticated automated methods dedicated to medical diagnostic laboratories for routine work may not keep up with taxonomic changes and the new species described. The identification of new species at diagnostic laboratories is challenging but of crucial importance due to the fact that new species are revealed to be potentially harmful to humans as strains belonging to *C. diphtheriae* complex might change into toxin-producing pathogens and cause serious potentially fatal infections among non-vaccinated individuals, but also among vaccinated individuals who did not receive a booster dose of anti-diphtheria vaccine. 

## Figures and Tables

**Table 1:** Differentiation of the *C. diphtheriae* biovars on the basis of colony morphology on primary media and biochemical properties [85].

*C. diphtheriae* Biovar	Blood Agar	Hoyle’s Tellurite Agar	Lipophilism	Nitrate Reduction	Ability to Utilize Glycogen
Gravis	non-hemolytic	dull, grey/black, opaque colonies, 1.5–2.0 mm in diameter, matt surface, friable, tending to break into small segments when touched with a straight wire	−	+	+
Mitis	colonies may exhibit a small zone of β-haemolysis	grey/black, opaque colonies, 1.5–2.0 mm in diameter, entire edge and glossy smooth surface; size variation is common	−	+	−
Intermedius	colonies exhibit a small zone of β-haemolysis	small, grey/black, shiny surface, discrete, translucent colonies, 0.5–1.0 mm in diameter	+	+	−
Belfanti	colonies may exhibit a small zone of β-haemolysis	grey/black, opaque colonies, 1.5–2.0 mm in diameter, entire edge and glossy smooth surface, size variation is common	−	−	−

Colony morphology after 24 h of aerobic incubation at 35–37 °C. + positive; − negative.

## Data Availability

Not applicable.
